# Disseminated *Mycobacterium chimaera* infection in a patient with adult-onset immunodeficiency syndrome: case report

**DOI:** 10.1186/s12879-022-07656-0

**Published:** 2022-08-01

**Authors:** Yi-Fu Lin, Tai-Fen Lee, Un-In Wu, Chun-Fu Huang, Aristine Cheng, Kuan-Yin Lin, Chien-Ching Hung

**Affiliations:** 1 Department of Internal Medicine, National Taiwan University Hospital and National Taiwan University College of Medicine, 7 Chung-Shan South Road, Taipei, Taiwan; 2 Department of Laboratory Medicine, National Taiwan University Hospital and National Taiwan University College of Medicine, Taipei, Taiwan; 3 Department of Medicine, National Taiwan University Cancer Center, Taipei, Taiwan; 4 Department of Tropical Medicine and Parasitology, National Taiwan University College of Medicine, Taipei, Taiwan

**Keywords:** Anti-interferon-γ antibodies, Genetic predisposition, Immunosuppression, Non-tuberculous mycobacteria, *Mycobacterium avium* complex

## Abstract

**Background:**

Patients with adult-onset immunodeficiency syndrome due to anti-interferon-γ autoantibodies (AIGAs) are susceptible to disseminated *Mycobacterium avium* complex (MAC) infections. *M. chimaera,* a newly identified MAC species, is distinguished from the others due to the reduced virulence. Previous cases of disseminated *M. chimaera* infection have been linked to cardiothoracic surgery. Reports of disseminated *M. chimaera* in patients without a history of cardiothoracic surgery are rare.

**Case presentation:**

A 57-year-old Asian man, previously healthy, presented with fever, dry cough, exertional dyspnea, and decreased appetite. The delayed resolution of pneumonia despite antibiotic treatment prompted further imaging studies and biopsies from the lung and lymph node. The fluorodeoxyglucose positron emission tomography/computed tomography (FDG-PET/CT) demonstrated intense uptake in lung consolidations and diffuse lymphadenopathy. Cultures of the specimens obtained from sputum, blood, stool, lung tissue, and lymph node grew *M. chimaera*. Further immunological evaluation disclosed the presence of neutralizing AIGAs, which possibly led to acquired immunodeficiency and disseminated *M. chimaera* infection.

**Conclusions:**

We herein present the first case of adult-onset immunodeficiency due to AIGAs complicated with disseminated *M. chimaera* infection. Further immunological evaluation, including AIGAs, may be warranted in otherwise healthy patients who present with disseminated mycobacterial infection.

**Supplementary Information:**

The online version contains supplementary material available at 10.1186/s12879-022-07656-0.

## Background

Adult-onset immunodeficiency syndrome due to anti-interferon-γ autoantibodies (AIGAs) is associated with susceptibility to selective infections [1]. Previous studies demonstrated that a high proportion of infections in patients with AIGAs were caused by mycobacteria (85.5%); among them, *Mycobacterium avium* complex (MAC) and *M. abscessus* were most commonly reported [2]. Disseminated MAC infection characteristically affects immunocompromised patients; however, *M. chimaera* is a newly described slow-growing non-tuberculous mycobacterium (NTM) belonging to the MAC and distinguished from the other MAC species due to the relatively reduced virulence. *M. chimaera* has been reported to cause pulmonary infection, recently linked to infections after cardiothoracic surgery and outbreaks attributed to contaminated heater-cooler devices (HCDs) [3, 4]. The clinical presentations of *M. chimaera* infection include pulmonary diseases and extrapulmonary manifestations, including surgical site infection, prosthetic valve endocarditis, vascular graft infection, arthritis, osteomyelitis, and disseminated infection [5]. Although disseminated MAC infection disproportionally affects immunocompromised hosts, disseminated *M. chimaera* infection has more commonly occurred in patients undergoing cardiothoracic surgery and rarely been reported in patients with immune dysfunction [4, 6, 7]. We herein report the first case of adult-onset immunodeficiency due to AIGAs complicated with disseminated *M. chimaera* infection.

## Case presentation

**Fig. 1:**
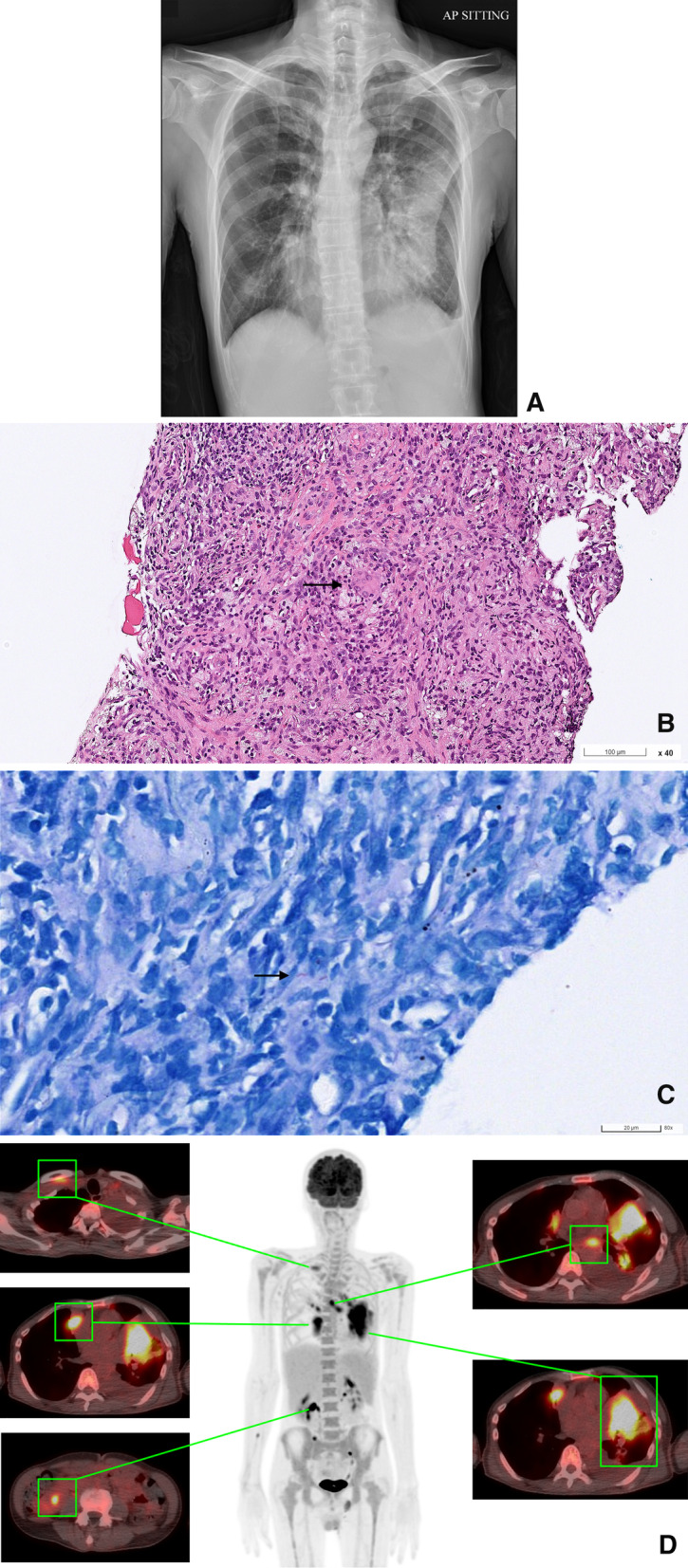
Imaging and pathology findings. **A** Chest radiograph showed irregular opacities at the left lower lung and blunted left costophrenic angle. **B**, **C** Hematoxylin and eosin and acid-fast stained lung sections revealed granulomatous inflammation with the presence of acid-fast bacilli. **D** FDG-PET/CT showed intense FDG hypermetabolism at bilateral lungs, especially LUL (SUV_max_, 12.3) and multiple hot areas at bilateral mediastinal nodes (SUV_max_, 8.6), bilateral supraclavicular regions (SUV_max_, 4.9), right mesenteric region (SUV_max_, 8.9) and left para-sternal node (SUV_max_, 3.1). FDG PET-CT, fluorodeoxyglucose positron emission tomography-computed tomography; SUV_max_, maximum standardized uptake value

A 57-year-old Malaysian Chinese man, previously healthy, presented to our hospital with fever and exertional dyspnea for 1 week. One month before the current presentation, he developed dry cough and progressively decreased appetite. He had undergone appendectomy in his childhood but had no other systemic disease. There was no known family history of immunodeficiency or exposure to immunosuppressants before disease onset. On assessment, he was hemodynamically stable with the heart rate of 111 beats per minute, the blood pressure 106/60 mmHg, the respirations 18 breaths per minute, and the temperature 38.4 °C. Physical examination revealed decreased breath sounds on the left lung and a swollen lymph node in the right neck. Laboratory evaluations showed leukocytosis (18,690 cells/μL) with neutrophilia (89%), anemia (7.9 g/dL), thrombocytosis (736,000 cells/μL), elevated C-reactive protein (15.81 mg/dL) and elevated alkaline phosphatase (145 U/L). A chest radiograph and computed tomography (CT) demonstrated multifocal patchy consolidations, bilateral pleural effusion, and enlarged subcarinal lymph nodes (Fig. 1A). A pleural fluid analysis revealed neutrophil-predominant exudates.

Ceftriaxone was administered intravenously at a dose of 1000 mg twice daily for 10 days, but his clinical condition and chest radiographs did not improve. The delayed resolution of pneumonia prompted a sonography-guided lung biopsy, in which pathology disclosed granulomatous inflammation with the presence of acid-fast bacilli (Fig. 1B and C). Colonoscopy showed multiple shallow cecal ulcers. The fluorodeoxyglucose-positron emission tomography/CT (FDG-PET/CT) for assessing the extent and severity of disease activity demonstrated intense uptake in lung consolidations and lymph nodes at bilateral supraclavicular, bilateral mediastinal, left para-sternal, and right mesenteric regions (Fig. 1D). Subsequently, the cultures of specimens obtained from sputum, blood, stool, lung tissue, and cervical lymph node revealed *M. chimaera*. A diagnosis of disseminated *M. chimaera* infection was made, and he was initially treated with amikacin (750 mg infusion once daily), rifabutin (300 mg orally once daily), azithromycin (500 mg orally once daily), and ethambutol (800 mg orally once daily). Three days after initiation of anti-mycobacterial therapy, the low-grade fever, leukocytosis, and elevated alkaline phosphatase persisted; and therefore, moxifloxacin (400 mg orally once daily) was added to the treatment regimen. The results of antimicrobial susceptibility testing are summarized in Additional file 1.

The investigations of immune deficiency showed no evidence of human immunodeficiency virus (HIV) infection, elevated immunoglobulin G (IgG) levels, and normal counts of lymphocyte subsets and monocyte. He had a high anti-nuclear antibody titer of 1:1280 with a fine speckled pattern, but did not meet the criteria of autoimmune diseases due to the absence of associated symptoms and specific extractable nuclear antigens. He tested positive for neutralizing AIGAs, with a plasma concentration of 901 μg/ml (reference value, 15.78 μg/ml). The methodologic details on AIGAs enzyme-linked immunosorbent assay (ELISA) are shown in Additional file 1. The patient carried the human leukocyte antigen (HLA) risk alleles (HLA-DRB1*16:02, HLA-DQB1*05:02), which were recently recognized to confer genetic predisposition to AIGAs production in populations originatied from Southeast Asia [8]. His symptoms resolved gradually during subsequent follow-up, and the chest CT and colonoscopy one month later showed significant improvement in the consolidations and healing ulcers, respectively. Amikacin, rifabutin, and azithromycin were subsequently discontinued 1, 2, and 4 months later, respectively. As of 30 June, 2022, he had received ethambutol and moxifloxacin for 7 months. Since the follow-up chest radiography and laboratory data showed significant improvement, FDG-PET/CT will be scheduled 12 months after anti-mycobacterial therapy to guide the treatment duration.

## Discussion and conclusions

*Mycobacterium chimaera* has been shown to be of low virulence, but could cause pulmonary infection as a consequence of colonization to the airways in patients with immunodeficiency or pre-existing lung conditions [5]. The treatment outcomes of *M. chimaera* lung disease were reported to be suboptimal despite the combination use of long-term antimicrobial agents [9]. On the other hand, disseminated *M. chimaera* infection had been scarcely described until 2013, when the first two cases were reported in patients with prosthetic valve endocarditis and bloodstream infection due to *M. chimaera* after open-chest cardiac surgery. Afterwards, more cases were identified in the prolonged outbreak, which was linked to a source of contaminated water in HCDs connected to the cardiopulmonary bypass. The increased use of contaminated HCDs has led to HCD-associated *M. chimaera* outbreaks globally since 2015, with more than 120 cases of *M. chimaera* infections reported following cardiac surgery in the literature [10]. With a long latency period (median, 21 months) between cardiac surgery and symptoms, the mortality rate of post-cardiac surgery *M. chimaera* infection was remarkably high (50%) [11]. To evaluate the disease extent and detect recurrence, FDG-PET/CT imaging is a crucial diagnostic tool and should be considered early in patients with disseminated *M. chimaera* infection [12].

Apart from systemic infection caused by contaminated HCDs, only two cases developed disseminated *M. chimaera* infection attributable to their immunodeficiency (Table 1) [6, 7]. One patient received prednisolone and hydroxychloroquine for systemic lupus erythematosus, and the other patient underwent chemotherapy for diffuse large B-cell lymphoma. Although both cases had underlying immune dysregulation and received immunosuppressants, disseminated *M. chimaera* infection was successfully treated with prolonged courses of antibiotics. Considering a rare occurrence of disseminated *M. chimaera* infection in patients without a history of cardiothoracic surgery, investigations to identify immune deficiency was performed in our patient. Adult-onset immunodeficiency syndrome due to AIGAs has been extensively described in East Asian populations [2], which prompted testing for AIGAs in this case.

**Table 1:** Summary of disseminated *Mycobacterium chimaera* infection in patients without a history of cardiothoracic surgery in the literature

References/country	Age/gender	Underlying disease	Manifestations	IGRA testing	Treatment regimen	Treatment duration	Outcome
Moutsoglou et al. [6]/USA	75/female	Systemic lupus erythematosus, receiving prednisone and hydroxychloroquine	Vertebral osteomyelitis; calf wound infection	Indeterminate	Clarithromycin, rifampin, ethambutol	Prolonged	Successfully treated
de Melo Carvalho et al. [7]/Portugal	57/male	Diffuse large B-cell lymphoma, receiving R-CHOP	Bloodstream infection; bone marrow involvement; pneumonia; hepatosplenomegaly	Negative	Clarithromycin, rifabutin, ethambutol, moxifloxacin	12 months	Successfully treated
Present case/Taiwan	57/male	Adult-onset immunodeficiency syndrome due to anti-interferon-γ autoantibodies	Bloodstream infection; pneumonia; lymphadenopathy	NA	Amikacin, azithromycin, rifabutin, ethambutol, moxifloxacin	Prolonged	Successfully treated

^*^R-CHOP, rituximab, cyclophosphamide, doxorubicin, vincristine and prednisone

Despite geographically varied distribution of NTM species, the dominant NTM species resulting in disseminated infection among patients with AIGAs is MAC (30.7%) [2]. In addition to the main species of MAC (*M. avium* and *M. intracellulare*), new MAC species (*M. colombiense* and *M. mantenii*) have been reported to cause disseminated infection in patients with AIGAs [13–15]. *M. chimaera*, a recently identified species of MAC, has a lower virulence than the two original species of MAC [3]. We present the first case of disseminated *M. chimaera* infection in a patient with AIGAs; nevertheless, systemic infection caused by *M. chimaera* among patients with AIGAs may be under-diagnosed due to inaccessibility to AIGAs ELISA and identification of MAC species. To reduce misdiagnosis, the commercially available interferon-γ release assay (IGRA) could be used in the clinical setting without readily accessible AIGAs ELISA. An indeterminate IGRA result may indicate the presence of neutralizing AIGAs in a patient with disseminated NTM infection [16, 17].

The present case highlights that disseminated *M. chimaera* infection can also occur in patients with adult-onset immunodeficiency syndrome due to AIGAs. Early recognition and immunological evaluation in otherwise healthy patients who present with disseminated mycobacterial infection are essential to improving outcomes.

## Supplementary Information

**Additional file 1.** Supplementary material.

## Data Availability

All data generated or analyzed during this study is included in this published article.

## Abbreviations

AIGAs: Anti-interferon-γ autoantibodies
CT: Computed tomography
ELISA: Enzyme-linked immunosorbent assay
FDG: Fluorodeoxyglucose
HIV: Human immunodeficiency virus
HCDs: Heater–cooler devices
HLA: Human leukocyte antigen
IgG: Immunoglobulin G
IGRA: Interferon-γ release assay
MAC: *Mycobacterium avium* Complex
NTM: Non-tuberculous mycobacterium
PET: Positron emission tomography
SUV_max_: Maximum standardized uptake value
