# Supplementary material for: Disseminated Mycobacterium chimaera infection in a patient with adult-onset immunodeficiency syndrome: case report
Source: BMC Infect Dis. 2022 Aug 1;22:665. doi: 10.1186/s12879-022-07656-0 (PMC9344727; doi:10.1186/s12879-022-07656-0)
Supplement: Supplementary file 1 — Additional file 1. Supplementary material. [file 12879_2022_7656_MOESM1_ESM.docx]

**Supplementary material**

**Culture and identification for *Mycobacterium chimaera***

Specimens tested for mycobacteria were homogenized and decontaminated by the N-Acetyl L-cysteine (NALC)/Sodium hydroxide (NaOH) method. Specimens were inoculated on solid medium (Löwenstein-Jensen; bioMérieux, Marcy l'Etoile, France) and liquid medium (mycobacteria growth indicator tube [MGIT]), with incubating at 35 to 37°C for up to 6 weeks. The specimens in LJ medium were incubated in a 5% CO2 incubator and examined once a week. The MGIT tubes were placed in the BACTEC MGIT 960 system (Becton Dickinson, Sparks, MD, USA), which was a fluorescence-based detection system. The culture-positive specimens were subcultured onto Middlebrook 7H11 selective agar. After protein extraction, we deposited samples on the matrix-assisted laser desorption/ionization-time of flight (MALDI-TOF) mass spectrometry (MS) target plate (Bruker Daltonics, Bremen, Germany). The target plate was inserted into the MALDI-TOF microflex LT (Bruker Daltonics), and spectra were obtained over a mass/charge (m/z) ratio of 2,000 to 20,000 Da using Mycobacteria Library v4.0 database. The software assigned a score from 0 to 3 and classified results into 3 categories: reliable (species level; ≥2), probable (genus level; 1.7 to 1.9), and nonidentifiable (<1.7). A score of ≥1.7 was used as the acceptable cutoff for specimen identification.

**Antimicrobial susceptibility testing of the *Mycobacterium chimaera* isolate from this patient**

| **Antibiotics** | **MIC (mg/L)^a^** | **Susceptibility category^b^** |
| --- | --- | --- |
| Clarithromycin | 4 | S |
| Rifabutin | 2 | NA |
| Rifampicin | >8 | NA |
| Ethambutol | 8 | NA |
| Amikacin | 16 | S |
| Streptomycin | >64 | NA |
| Moxifloxacin | 8 | R (tentative) |
| Ciprofloxacin | >16 | NA |
| Isoniazid | >8 | NA |
| Linezolid | 32 | R (tentative) |
| Trimethoprim/Sulfamethoxazole | 8/152 | NA |
| Doxycycline | >16 | NA |
| Ethionamide | 20 | NA |

^a^Antimicrobial susceptibility testing was performed using the SLOWMYCOI Sensititre™ panel (Trek Diagnostic Systems, East Grinstead, UK) according to CLSI protocol M24-A3.

^b^Interpretive criteria were used according to CLSI Standard M24-A3 for clarithromycin (S ≤8 mg/L, I 16 mg/L, R ≥32 mg/L), amikacin (S ≤16 mg/L, I 32 mg/L, R ≥64 mg/L), moxifloxacin (tentative; S ≤ 1 mg/L, I 2 mg/L, R ≥4 mg/L), and linezolid (tentative; S ≤8 mg/L, I 16 mg/L, R ≥32 mg/L).

**Abbreviations:** I, intermediate; MIC, minimum inhibitory concentration; NA, not available; R, resistant; S, susceptible.

**Anti-interferon-γ antibodies (AIGAs) enzyme-linked-immunosorbent assay (ELISA)**

Microtiter plates were coated with 10 μg/mL of interferon-γ (R&D Systems, Minneapolis, MN) per well, incubated at 4°C overnight, and then washed 3 times with phosphate-buffered saline solution (PBST) (0.1% Tween 20/PBS, Sigma, St Louis, MO). Recombinant mouse anti-human interferon-γ antibodies (R&D Systems) were used as standards with serial dilution. The diluted plasma, diluent buffer (blank), standards, positive and negative controls were added in duplicate to each well and incubated at room temperature for 3 hours. After washing 3 times with PBST, the standards were incubated with horseradish peroxidase (HRP)-conjugated donkey anti-mouse immunoglobulin G (IgG) (Thermo Scientific, Rockford, IL, USA), while the patient plasma and controls wells were incubated with HRP-conjugated sheep anti-human IgG (Thermo Scientific) for 1.5 hours. After 3 washes, 3,3',5,5'-Tetramethylbenzidine (TMB) substrate (Thermo Scientific Rockford, IL) was added to each well and incubated for 30 min, and stopping solution (Thermo Scientific, Rockford, IL) was added before determining the optical density at 450–550 nm. The normal range for the anti-interferon-γ antibodies concentration was defined by the 99th percentile for 100 healthy volunteers. Concentrations higher than the normal range were classified as positive for anti-interferon-γ antibodies.
